# The Patient Experience of Acute Lymphoblastic Leukemia and Its Treatment: Social Media Review

**DOI:** 10.2196/39852

**Published:** 2023-05-01

**Authors:** Rebecca Crawford, Slaven Sikirica, Ross Morrison, Joseph C Cappelleri, Alexander Russell-Smith, Richa Shah, Helen Chadwick, Lynda Doward

**Affiliations:** 1 Research Triangle Institute Health Solutions Manchester United Kingdom; 2 Pfizer Inc New York City, NY United States

**Keywords:** acute lymphoblastic leukemia, health-related quality of life, qualitative research, social media, leukemia, lymphoblastic, adult, disease, treatment, therapy, symptoms, independence, functioning, social, well-being, emotional

## Abstract

**Background:**

Adult patients with acute lymphoblastic leukemia (ALL) report substantial disease- and treatment-related impacts on their health-related quality of life (HRQOL). Patient-reported information (PRI) shared on social media may provide a distinct opportunity to understand the patient experience outside of formal research contexts and help inform the development of novel therapies.

**Objective:**

This qualitative social media review aimed to assess PRI shared on social media websites to gain a better understanding of the symptom, HRQOL, and treatment impacts on individuals with ALL.

**Methods:**

We identified English-language posts on 3 patient advocacy websites (Patient Power, The Patient Story, and Leukaemia Care) and YouTube that included PRI about experiences with ALL or ALL treatments shared by adults (aged ≥18 years) with a self-reported ALL diagnosis. Patients’ demographic and disease characteristics were extracted from posts (where available), and the posts were analyzed thematically. A network analysis was conducted to delineate possible associations among ALL symptoms, HRQOL impacts, and treatment-related symptoms and impacts.

**Results:**

Of the 935 social media posts identified, 63 (7%) met the review criteria, including 40 (63%) videos, 5 (8%) comments posted in response to videos, and 18 (29%) blog posts. The 63 posts were contributed by 41 patients comprised of 21 (51%) males, 18 females (44%), and 2 (5%) whose gender was not reported. Among the patients, 13 (32%) contributed >1 source of data. Fatigue (n=20, 49%), shortness of breath (n=13, 32%), and bruising (n=12, 29%) were the symptoms prior to treatment most frequently discussed by patients. Patients also reported impacts on personal relationships (n=26, 63%), psychological and emotional well-being (n=25, 61%), and work (n=16, 39%). Although inpatient treatment reportedly restricted patients’ independence and social functioning, it also provided a few patients with a sense of safety. Patients frequently relied on their doctors to drive their treatment decisions but were also influenced by family members. The network analysis indicated that disease-related symptoms were primarily associated with patients’ physical functioning, activities of daily living, and ability to work, while treatment-related symptoms were primarily associated with emotional well-being.

**Conclusions:**

This social media review explored PRI through a thematic analysis of patient-contributed content on patient advocacy websites and YouTube to identify and contextualize emergent themes in patient experiences with ALL and its treatments. To our knowledge, this is the first study to leverage this novel tool to generate new insights into patients’ experiences with ALL. Patients’ social media posts suggest that inpatient care for ALL is associated with restricted independence and social functioning. However, inpatient care also provided a sense of safety for some patients. Studies such as this one that capture patients’ experiences in their own words are valuable tools to further our knowledge of patient outcomes with ALL.

## Introduction

Acute lymphoblastic leukemia (ALL) is an aggressive cancer of the blood and bone marrow that rapidly progresses and affects immature blood cells rather than mature ones [1]. ALL is the most common childhood cancer (ie, in patients under 18 years of age, the median age of diagnosis is 15 years), but it also accounts for approximately 20% of adult leukemias [2,3]. Childhood ALL has a cure rate as high as 90%, but the cure rate for adults is substantially lower, ranging from 20% to 40% [1,3,4].

Along with a poor prognosis, patients with ALL experience a significant symptom burden that impacts their physical, social, and emotional functioning [5,6]. This symptom burden is often compounded by significant chemotherapy-associated toxicity as well as frequent and extended hospital stays [1,4,7]. As novel therapies for adult ALL are developed and their uptake increases, a greater insight into patients’ experiences with ALL and the impact of ALL symptoms and treatments on patients’ health-related quality of life (HRQOL) is needed.

Patient-reported information (PRI) uploaded to social media websites provides a rich source of unsolicited data to facilitate a better understanding of how patients experience a disease and its treatment outside of the formal research context [8]. PRI data include information shared on social media as either single micronarratives (eg, video logs) or interactive micronarratives generated as part of discussions with other patients, caregivers, or stakeholders (eg, chat room discussions [8]). Both the US Food and Drug Administration and European Medicines Agency encourage the exploration of social media as a tool to better understand patient perspectives on disease symptoms and impacts [9,10].

Accordingly, this social media review explored PRI through a thematic analysis of patient-contributed content on patient advocacy websites and YouTube to identify and contextualize emergent themes in patient experiences with ALL and its treatments. To our knowledge, this is the first study to leverage this novel tool to generate new insights into patients’ experiences with ALL.

## Methods

### Search Strategy and Data Sources

The social media review was conducted in October 2020. A pragmatic Google search was performed by experienced qualitative researchers (authors RC, RM, and HC) to identify patient advocacy websites that hosted patient-contributed content. Google’s advanced search function was used to identify webpages that included any of the following key search terms: “acute lymphoblastic leukemia,” “acute lymphoblastic leukaemia,” “patient narratives,” “patient stories,” “patient advocacy,” and “patient organization.” The results were then reviewed to identify websites that might contain PRI describing the patient experience of ALL and its treatment, including patient ALL organization websites. PRI was defined as information reported by patients (or caregivers) relating to their experience of disease and its treatment outside a formal research context [8]. The contents of the websites were reviewed to ascertain whether they contained relevant PRI, and websites without relevant PRI were excluded from the review. The Research Triangle Institute (RTI) Health Solutions staff who reviewed the website content were both male and female researchers who had experience with qualitative research methods.

Five relevant websites were identified: Cure Today, Patient Power, Patients Rising, The Patient Story, and Leukaemia Care [11-15]. These patient advocacy websites provide information and support for people affected by cancer and include interviews conducted with patients, caregivers, and patient advocates that focus on specific cancers and treatments. Therefore, these websites were considered to contain the relevant PRI for data collection. Of the 5 websites, 3 (60%) contained PRI related to the patient experience of ALL and ALL treatments (Patient Power, The Patient Story, and Leukaemia Care). Permission was sought from the websites to use their content for this study. A YouTube search using similar search terms as the Google search for identifying websites was also conducted to identify additional ALL-related PRI. YouTube is a global online platform where registered users can easily upload and share videos. Videos uploaded with “public” privacy settings, which can be viewed by any internet user, were the focus of this search.

The review of the patient advocacy websites and YouTube targeted PRI uploaded by social media contributors with a self-reported diagnosis of ALL who discussed their experience with ALL and/or its treatment. Posts were considered eligible for inclusion if they were shared by adults (≥18 years of age) with a self-reported ALL diagnosis, if the adult patient contributed the PRI themselves and not by a proxy (eg, caregiver, physician, or relative), if the post was in English, and if the content was relevant to the patient experience of ALL and/or its treatment. All video footage and blog posts were manually reviewed by RTI-trained researchers to determine eligibility for inclusion in the review. Specifically, 2 RTI researchers reviewed the blogs/posts and created a data record that included search terms, date of search, and the number of views. They also noted the PRI associated with symptoms, HRQOL impacts, and demographics. Blog posts were excluded if they did not meet the following inclusion criteria: not specific to the target disease (ie, ALL), adult patient–focused, written in English, and patient report.

### Data Extraction and Analysis

#### Patient Characteristics

Patients’ demographic information (ie, age and sex) and disease characteristics were extracted from social media posts and were assumed to represent their characteristics at the time they uploaded the post. The posts were transcribed, and key data from the posts were extracted into a data record by 1 of the 3 RTI Health Solutions researchers (authors HC, RM, and RC). Since PRI exists outside of the traditional research context, key demographic and disease characteristics were not always available. Where possible, the demographic data available in posts were cross-checked with the patient’s username/handle on the same website, their profile associated with the post, or a photograph of themselves that they uploaded to the website. The number of distinct social media posts that each patient contributed was recorded, as well as key parameters for video data, such as upload date, video duration, and type of video publisher/poster (eg, independent patient, medical organization, or pharmaceutical company). Individual posts were cross-checked, when possible, to identify whether the same patient had contributed to more than 1 social media post (eg, if they contributed to both a blog post and a video).

#### Thematic Analysis

A thematic analysis of the aggregated PRI data extracted from the social media posts was conducted. In this type of analysis, a theme is described as content that captures data relevant to the research question and appears as a patterned response [16]. Specifically, relevant sections from the blog/posts were transcribed, and key themes such as symptoms and HRQOL impact (ie, physical, emotional, relationships, social life, activities of daily living, and work) related to the patient experience of ALL and themes related to treatment, such as treatment history, current treatment, treatment expectations, preference, side effects, impact, time spent receiving treatment, and decision-making, were identified and summarized with quotes. All data were coded by 1 of the 3 RTI researchers (authors HC, RC, and RM) into the key theme categories of symptom, HRQOL, and treatment impacts.

**Figure 1:**
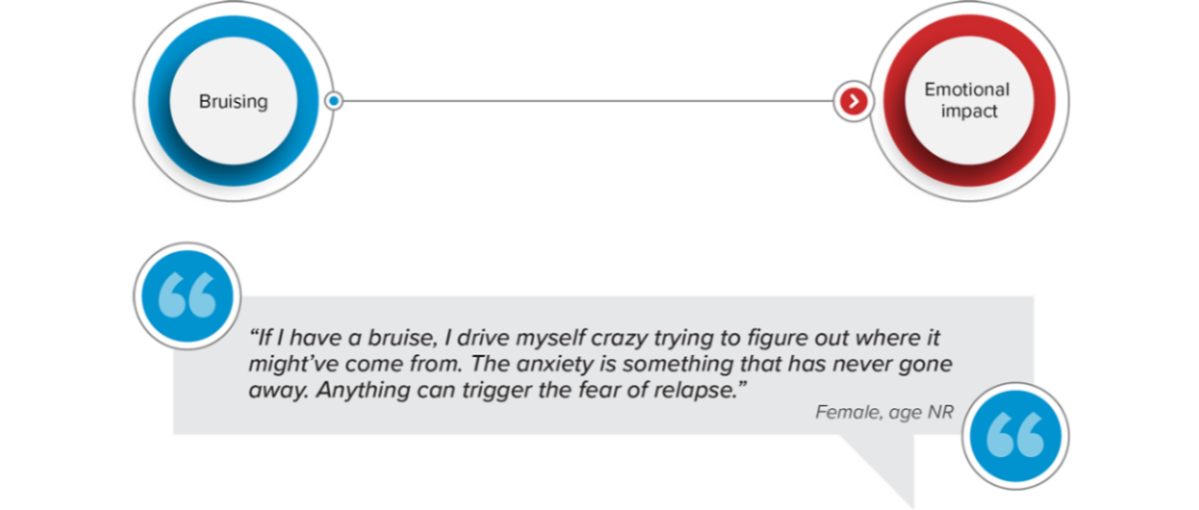
Example of network analysis relationship. NR: not reported.

A network analysis was also conducted to identify potential associations between ALL symptoms, HRQOL impacts, and treatment-related symptoms and impacts. The analysis was informed by the network approach to psychopathology, which conceptualizes mental disorders as a network of interacting symptoms [17]. In the analysis, nodes represented distinct ALL symptoms, HRQOL impacts, and treatment-related symptoms and impacts. Edges represented patient-indicated associations between 2 concepts. The edges were directional to indicate sequential associations (eg, frequent bruising preceded anxiety). To illustrate an example (Figure 1), the nodes for ALL symptoms represent 1 theme, the HRQOL impacts nodes represent a second theme, and the edges that connect the 2 themes demonstrate how they could be related or associated based on patient-reported experiences with ALL.

### Ethical Considerations

The RTI International Institutional Review Board determined that this study did not constitute research with human participants (STUDY00021294). The contributor quotes used to illustrate the key findings from the social media review are deidentified to maintain contributor confidentiality. No relationship existed between the researchers and the patients prior to conducting this study.

## Results

### Social Media Posts

**Figure 2:**
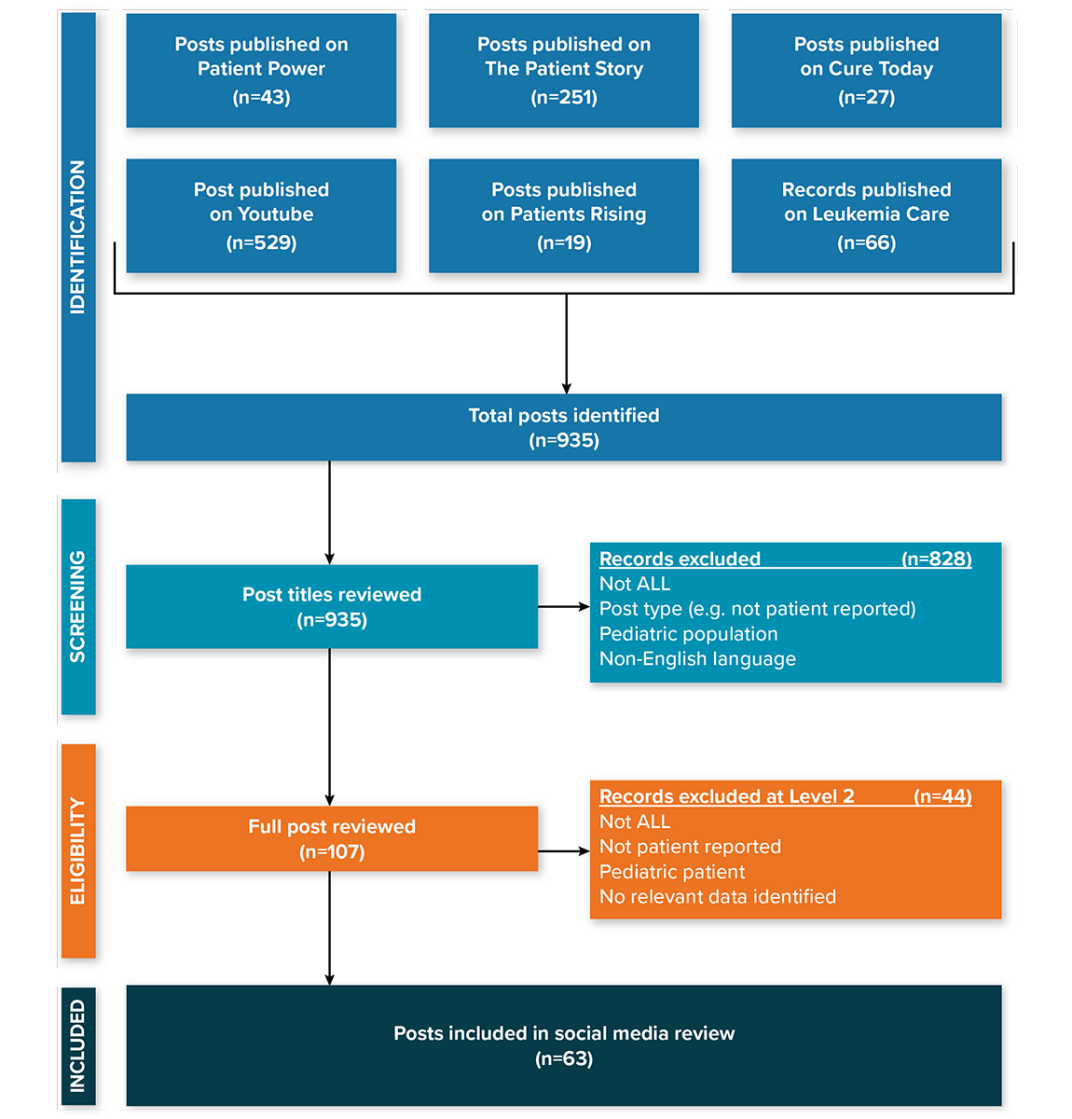
Social media post identification flowchart. ALL: acute lymphoblastic leukemia.

A total of 935 social media posts were identified and assessed in terms of the prespecified review criteria. Of the 935 posts, 63 (7%) were included in the final review from Leukemia Care (n=12, 19%), The Patient Story (n=6, 10%), Patient Power (n=4, 6%), and YouTube (n=41, 65%) (Figure 2, Multimedia Appendix 1). The 63 posts included 40 videos totaling 6 hours, 5 minutes, and 27 seconds of footage (mean 9 minutes, 8 seconds; range, 58 seconds to 1 hour, 14 minutes, and 12 seconds); 5 comments posted by patients on 3 of the videos; and 18 blog posts. The posts were uploaded between 2014 and 2020, with most (n=55, 87%) uploaded in 2018 or later.

### Patient Characteristics

The 63 social media posts included PRI from 41 individual patients. Table 1 provides the sample characteristics (gender, age range, and country or origin of the contributor post). Among the 41 patients, 13 (32%) contributed to more than 1 post. Most (n=34, 83%) of the patients were identified as located in either the United States (n=19, 46%) or the United Kingdom (n=15, 37%). The remaining 7 patients included 3 patients located in Australia (n=1, 2%), Canada (n=1, 2%), and South Africa (n=1, 2%) and 4 (10%) whose locations were indeterminable based on the available data (all 4 provided comments on YouTube videos). Identities were cross-checked against other content the patients had uploaded to the same website (ie, username/handle, profile, or photograph) for all but 3 patients (7%) who posted relevant PRI as comments on YouTube videos. Approximately half (n=21, 51%) of the patients were male, and 2 (5%) did not report their gender. Age was available for 26 (63%) of the 41 individual patients and ranged from 19 to 59 years.

**Table 1:** Summary of social media contributor sample characteristics^a^.

Contributor characteristics		Value, n (%)
**Gender**		
	Male	21 (51)
	Female	18 (44)
	Not reported	2 (5)
**Age (years) at SM^b^ post**		
	18 to <30	13 (32)
	30 to <40	11 (27)
	40 to <50	1 (2)
	≥50	1 (2)
	Not reported	15 (37)
**Country of origin**		
	United States	19 (46)
	United Kingdom	15 (37)
	Australia	1 (2)
	Canada	1 (2)
	South Africa	1 (2)
	Not reported	4 (10)

^a^Percentages are based on nonmissing data.

^b^SM: social media.

### Patient Symptom Experience and Impacts on HRQOL

Patients generally commented on the ALL symptoms they experienced before their initial diagnosis. They most frequently discussed fatigue (n=20, 49%), shortness of breath (n=13, 32%), and bruising (n=12, 29%) in their social media posts. Their symptoms impacted their physical functioning, such as difficulty climbing stairs or walking up an incline (n=5, 12%), getting out of bed (n=4, 10%), and walking short distances (n=3, 7%). Patients’ symptoms also interfered with their ability to live a normal life. Nearly half (n=16, 39%) of the patients reported impacts on their ability to work, and many (n=11, 27%) reported impacts on their usual daily activities, including difficulty with basic self-care (n=4, 10%), daily tasks such as chores and shopping (n=9, 22%), and hobbies and leisure activities (n=3, 7%). Two (5%) patients also noted limitations on their social functioning, such as having to practice social distancing at public venues (n=1, 2%) and missing social occasions (n=1, 2%).

Over half (n=26, 63%) of the patients reported a change in their relationships as a result of their ALL. For some (n=5, 12%) patients, their relationships reportedly improved and were strengthened by coping with their ALL symptoms. For others (n=2, 10%), their ALL symptoms were associated with a deterioration in their relationships. They lost touch with friends, and their relationships with their partners changed. For example, 1 patient described how she felt her ALL symptoms changed her relationship with her husband:

I felt like he was more my caregiver than my husband.

Female, age not reported

Over half (n=25, 61%) of the patients reported that their ALL had a deleterious impact on their psychological and emotional well-being. Patients reported a range of emotional and psychological impacts, including low mood (n=3, 7%), anxiety at the prospect of relapse (n=4, 10%), and loneliness (n=2, 5%).

Moreover, 2 (5%) patients described feeling betrayed by their body:

I felt a deep anger towards my own body; I felt betrayed by it.

Female, 27 years

Several also expressed fears about the future, such as mortality (n=4, 10%) and uncertainty about their ongoing disease (n=2, 10%). As 1 patient explained,

Not knowing at all what my life would look like was traumatizing for me.

Male, 33 years

### Patient Treatment Experience

Patients experienced a range of treatments for ALL, with nearly half (n=20, 49%) reporting experience with multiple types of treatment (Table 2). Fatigue (n=11, 27%), hair loss (n=11, 27%), and nausea (n=9, 22%) were the most frequently reported treatment-related side effects. These treatment side effects were reportedly often long lasting and had a negative impact on the patients’ physical functioning, including eating (n=4, 10%), fine motor skills (n=1, 2%), activities of daily living such as showering (n=1, 2%), and future reproductive abilities (n=1, 2%). These issues had a negative impact on the patients’ psychological well-being.

**Table 2:** Self-reported experience with acute lymphoblastic leukemia (ALL) treatment.

Treatment type	Self-reported experience, n (%)
Chemotherapy	31 (76)
Bone marrow transplant/stem cell transplant	16 (39)
Radiation therapy	5 (12)
Immunotherapy	4 (10)
Steroid treatment	4 (10)
Blood transfusion	1 (2)
Umbilical cord blood transplant	1 (2)

As 1 patient reported,

One night, um, my neuropathy and my hands were so bad and one of my…one of my kids wanted a peanut butter and jelly sandwich and to take the twist tie off the bread hurt so bad because my neuropathy was so bad, and I just broke down in the kitchen.

Female, 30 years

Furthermore, another patient concluded that the treatment for ALL was worse than the cancer itself:

The treatment made me feel worse than the cancer ever did. Eventually, I ended up fainting from exhaustion whilst attempting to shower.

Female, 27 years

Overall, three key themes emerged from the analysis of patients’ social media posts related to their treatment experience: (1) perceptions of inpatient treatment, (2) treatment expectations and preferences, and (3) treatment decision-making.

### Perceptions of Inpatient Treatment

Over a quarter (n=11, 27%) of patients reported their perceptions of inpatient treatment. Several (n=4, 10%) patients commented that inpatient treatment restricted their freedoms and independence. For example, 1 patient explained that when given the option, he chose to leave the hospital:

It was a situation where I could’ve stayed in the hospital, but I just want[ed] to be a little more independent and do things on my own. I much preferred that.

Male, 36 years

Some (n=4, 10%) patients also commented on how inpatient treatment impeded their social functioning:

I felt like I had lost total control of everything, not being able to see my family, friends, have fun. Nothing was normal anymore; the hospital became my new home.

Male, 34 years

The restrictive requirements of inpatient care were also a source of anxiety for 1 (2%) patient who was concerned about her ability to care for her children:

You can’t keep me here [the hospital], I just got here, I have no clothes, no toiletry bags, I didn’t get to say bye to my kids, I didn’t kiss them, who’s going to watch my kids?

Female, 30 years

In contrast to the negative patient perceptions of inpatient care, some (n=4, 10%) patients also highlighted the perceived benefits of inpatient treatment, such as its sense of safety. One patient reported that he felt afraid when leaving the hospital after a 6-week stay:

After 6 weeks in hospital, I could go home. I cried a bit at this point, as I was scared to leave the safety of the hospital.

Male, age not reported

Another patient expressed anxiety about losing the regularity of care provided in an inpatient setting:

If my consultant tells me he’ll see me again in 2 or 3 weeks, my first emotion is always disappointment, followed by apprehension at the prospect of going so long without a check-up.

Female, 27 years

One patient also appreciated having his treatment adherence controlled by the hospital staff:

When you’re in the hospital you don’t have to worry about anything like that [treatment adherence]. There’s going to be nurses that are going to be coming in…You pretty much do whatever they tell you to do.

Male, 36 years

### Treatment Expectations and Preferences

Over one-third (n=15, 37%) of patients discussed their treatment expectations and preferences in their social media posts. Patients reported that they often anticipated treatment side effects (n=4, 10%) but that the side effects were not always as severe as they expected (n=3, 7%). For example, 1 patient explained:

I want to tell leukemia and lymphoma patients to not be so afraid of transplant. I was super afraid.

Female, 29 years

Another patient described how his excitement about the potential positive outcome from a bone marrow transplant outweighed his concerns about the treatment burden:

I heard so many stories about having a [bone marrow] transplant, so I was excited to start the newest journey of my life, to get better, to be rid of ALL. It was a hard road ahead, but I had every faith.

Male, 34 years

In general, patients preferred treatments with minimal impact on their HRQOL. One patient preferred immunotherapy for this reason:

The beauty of immunotherapy is how little it affects your quality of life. Although side effects are possible, mine were minimal. [Male, 23 years] 

Another patient explained his desire for a treatment that allowed for an independent lifestyle:

I know that I’m getting treated, but at the same time, I have the freedom to coach my kids every day, to go about life, be able to drive my own car, and to go to work and be able to not have to have hospital food.

Male, 59 years

In contrast, 1 patient described the inevitable pain associated with chemotherapy:

[Intrathecal chemotherapy] was painful. That hurt. There’s nothing you can really do for it.

Female, 30 years

### Treatment Decision-making

Several (n=9, 22%) patients described their decision-making process in their social media posts. Of these patients, the majority (n=7, 78%) reported that their doctors drove their treatment decisions. As 1 patient explained,

Although things were always explained to us and I had to sign consent for treatments, I wasn’t really taking it in or paying real attention. I was just going along with it.

Female, age not reported

Another patient described his shock at the diagnosis and how this impacted his decision-making:

I was a little bit, um, obviously shocked because I didn’t know anything about leukemia…[I] didn’t know anything about chemotherapy or treatment, just sort of believed what the doctor told me.

Male, age not reported

However, a few (n=3, 7%) patients reported that their treatment decisions were also influenced by their parents:

She [mother] was also the one that was head honcho in all the research. She looked up everything. She looked up scientific studies on everything that was happening and all the treatments I was on.

Male, 23 years

### Network Analysis

**Figure 3:**
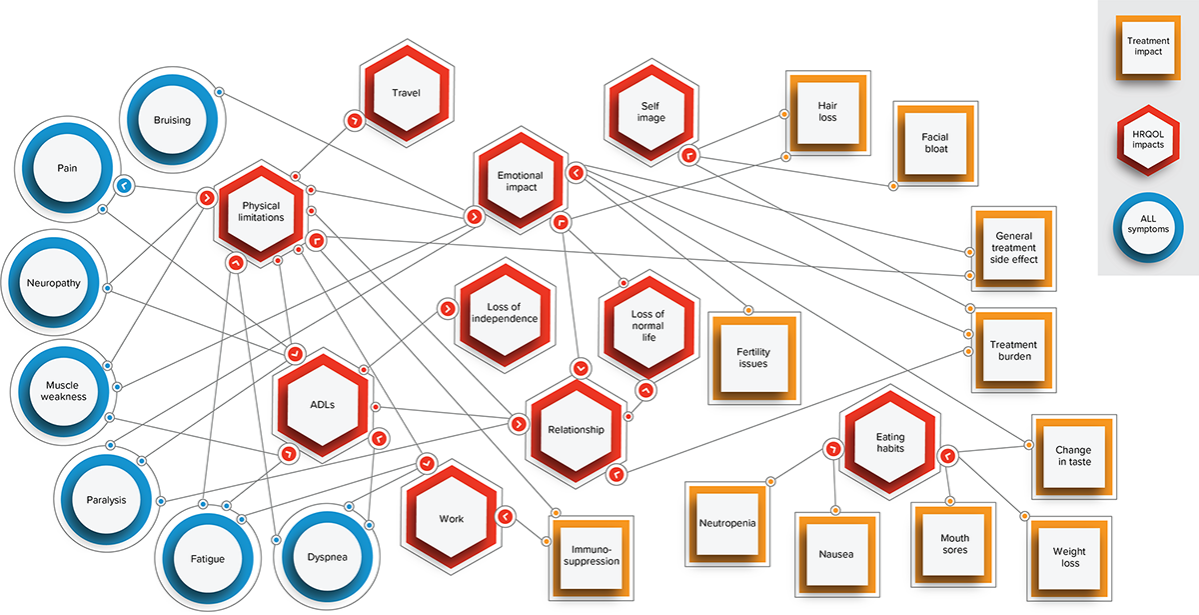
Acute lymphoblastic leukemia concept network. Concepts are connected based on patient-reported experiences. For example, bruising is connected to emotional impact based on the following quote: “If I have a bruise, I drive myself crazy trying to figure out where it might’ve come from. The anxiety is something that has never gone away. Anything can trigger the fear of relapse.” (Female, age not reported). ADL: activity of daily living; ALL: acute lymphoblastic leukemia; HRQOL: health-related quality of life.

Distinct associations among ALL symptoms, HRQOL impacts, and treatment-related symptoms and impacts were identified in the network analysis (Figure 3). ALL symptoms primarily affected patients’ physical functioning, activities of daily living, and ability to work. In contrast, treatment-related symptoms and impacts primarily affected patients’ emotional well-being. A cluster of treatment side effects (ie, neutropenia, change in taste, nausea, and mouth sores) was associated with changes in patients’ eating habits, which were in turn associated with weight loss. Three instances of this relationship were attributed to chemotherapy, while 1 instance was associated with a stem cell transplant. Physical limitations played the most central role in the HRQOL component of the network, impacting other aspects of patients’ HRQOL (ie, activities of daily living, work, travel, emotional well-being, and relationships).

## Discussion

### Principal Findings

This social media review explored PRI through a thematic analysis of patient-contributed content on patient advocacy websites and YouTube to identify and contextualize emergent themes in patient experiences with ALL and its treatments. To our knowledge, this is the first study to leverage social media websites to generate new insights into patients’ experiences with ALL. A network analysis of PRI also provided a distinct view of the connections among patients’ experiences with ALL symptoms, HRQOL impacts, and treatment-related symptoms and impacts. In our qualitative network analysis of patient-indicated associations among ALL symptoms, HRQOL impacts, and treatment-related symptoms and impacts, we found that ALL symptoms primarily affected patients’ physical functioning, activities of daily living, and ability to work, while treatment-related symptoms and impacts primarily affected patients’ emotional well-being. Overall, patients’ social media posts detailed the substantial HRQOL impacts they experienced due to their ALL symptoms and treatment side effects.

While studies of HRQOL among adult patients with ALL are limited, the substantial impacts of ALL on patients’ social, emotional, and physical functioning identified in this social media review are consistent with prior findings [5,6,18]. For example, Kantarjian et al [6] measured baseline symptom burden and functional impairment in patients with ALL using the European Organization for Research and Treatment of Cancer Quality of Life Questionnaire-Core Module (EORTC QLQ-C30) and found that fatigue, insomnia, pain, appetite loss, and dyspnea had the highest mean symptom scores (ie, worst symptom experience). In addition, a study evaluating HRQOL among adult ALL survivors found that pain and fatigue were the most commonly reported symptoms, and these symptoms were inversely correlated with social, cognitive, emotional, and physical function scores on the EORTC QLQ-C30 [18]. Similarly, our study found that patients frequently described experiencing ALL-related fatigue, difficulty breathing, and bruising in their social media posts. Patients also commented on their need for help from caregivers and how this impacted their relationships with their family members. These issues were identified in a recent review of peer-reviewed literature focused on the needs of family caregivers in the context of both adult and pediatric leukemia [19]. Given the complex care needs of adult patients with ALL and the substantial impacts on their HRQOL, there is an increasing focus on the need to balance treatment goals between achieving remission and maintaining or improving HRQOL [20]. Our findings further demonstrate this need for balance in the development of adult ALL therapies.

Three key themes emerged from our analysis of PRI about the treatment-related impacts of ALL: (1) patients’ perceptions of inpatient treatment, (2) their treatment expectations and preferences, and (3) their treatment decision-making. Most patients who commented on inpatient treatment felt that it restricted their independence and social functioning. Treatment-related hospitalization is common in adult ALL [21]. Therefore, it is important to understand how frequent inpatient stays impact patients’ HRQOL. For instance, patients’ social media posts demonstrated how extended hospital stays were particularly challenging for patients with children or grandchildren who relied on them for care. Interestingly, a few patients commented on the perceived benefits of inpatient treatment, noting that hospital routines and monitoring reassured them that they were receiving the necessary care.

As expected, patients who commented on their treatment preferences preferred treatments with minimal HRQOL impact. They expressed enthusiasm for treatments such as bone marrow transplant and immunotherapy, but they also commented on the inevitable pain of chemotherapy. When making treatment decisions, patients commented that they primarily deferred decision-making to their doctors. Their choice to defer treatment decisions to their doctor may have been influenced by their cognitive state (eg, shock, denial) at the time of diagnosis. For some patients, treatment decisions were also influenced by their parents, further highlighting the complex role of caregivers of adults living with ALL [19]. These 3 themes demonstrate the varied ways in which ALL treatments impact patients’ HRQOL and further highlight the need to minimize these impacts when developing ALL therapies.

Our analysis also showed that physical limitations were most central in the HRQOL component of the network, and they impacted patients’ ability to work, their relationships, and their emotional well-being. Elucidating the links among disease-related symptoms, treatment-related symptoms, and HRQOL impacts is critical to informing how clinicians treat patients, as illustrated by Wilson’s [22] conceptual model of the relationship between HRQOL and patient-reported outcome measures. Their model highlights the impact of symptoms, social context, and individual characteristics on functional status, which can then have downstream effects on the overall quality of life [22]. Therefore, our findings support the importance of minimizing the treatment burden for adult patients with ALL, as such treatment-related symptoms may have an additive effect alongside ALL-related symptoms that substantially impact patients’ HRQOL.

### Limitations

This social media review had several limitations worth noting. Social media data exist outside of the formal research context and are unregulated, so there is an inherent reliance on patient self-identification and self-report. There is also a risk of self-selection and publication bias. Patients who have a positive mindset may be more likely to submit their stories, and patient advocacy websites may be more likely to post inspirational content. There is also limited availability of patient demographic and clinical characteristics when relying on social media data. For example, age was not available for all patients included in the study, which limits our ability to identify potential age-related aspects of patients’ ALL experiences. Age may have been a key factor in determining how aggressive patients’ ALL treatment was since younger patients tend to receive more aggressive treatment than older patients. The social media data also lacked information on the stage of patients’ treatment journeys at the time of their post (eg, whether they were undergoing first-line treatment) as well as detailed information about other key clinical characteristics (eg, their Eastern Cooperative Oncology Group performance status). This is a new and growing field that requires strict adherence to terms and conditions for host websites, which can impact the type of information available to researchers. As the use of social media reviews to understand patient experiences becomes more common, guidelines will likely need to be developed to provide rigorous frameworks for these studies. Despite these limitations, this study provided valuable and rich insight into adult patients’ experiences with ALL through a novel analysis of PRI shared on social media. Patients reported that their ALL- and treatment-related symptoms had substantial impacts on their HRQOL, yet our findings indicate that ALL- and treatment-related symptoms impact different aspects of HRQOL. Treatments were burdensome for patients’ emotional well-being, while ALL symptoms primarily affected patients’ physical functioning. Inpatient treatment was particularly restrictive of their independence and social functioning but provided some patients with a sense of safety and security. Overall, patients desired treatments that minimized the impact on their HRQOL.

### Conclusion

The findings from this social media review suggest that inpatient care for ALL is associated with restricted independence and social functioning. However, inpatient care also provided a sense of safety for some patients. The PRI indicates that treatment- and ALL-related symptoms are associated with different HRQOL impacts, showing an explicit link between treatment-related symptoms and emotional well-being. A deeper understanding of patient experiences, especially disease-related symptoms, treatment-related symptoms, and HRQOL impacts, is critical to informing the development of new treatments and the utilization of current treatments. Studies such as this one that capture patients’ experiences in their own words are valuable tools to further our knowledge of patient outcomes with ALL. Information about this study in a plain language format is available in Multimedia Appendix 2.

## Appendix

Multimedia Appendix 1 Data sources.

Multimedia Appendix 2 Plain language summary.

## Abbreviations

ALL: acute lymphoblastic leukemia
EORTC QLQ-C30: European Organization for Research and Treatment of Cancer Quality of Life Questionnaire-Core Module
HRQOL: health-related quality of life
PRI: patient-reported information
RTI: Research Triangle Institute

## Data Availability

Data for this study were gathered from publicly available sources in accordance with their terms and conditions. Data sources used for this study can be found in Multimedia Appendix 1.
