# Correction: The Patient Experience of Acute Lymphoblastic Leukemia and Its Treatment: Social Media Review

**DOI:** 10.2196/54665

**Published:** 2023-12-20

**Authors:** Rebecca Crawford, Slaven Sikirica, Ross Morrison, Joseph C Cappelleri, Alexander Russell-Smith, Richa Shah, Helen Chadwick, Lynda Doward

**Affiliations:** 1 Research Triangle Institute Health Solutions Manchester United Kingdom; 2 Pfizer Inc New York City, NY United States

In “The Patient Experience of Acute Lymphoblastic Leukemia and Its Treatment: Social Media Review” (JMIR Cancer 2023;9:e39852), the authors made two updates.

A plain language summary was included as Multimedia Appendix 2.

The following sentence was added to the *Conclusion*, referring to the plain language summary:

Information about this study in a plain language format is available in Multimedia Appendix 2.

The correction will appear in the online version of the paper on the JMIR Publications website on December 20, 2023, together with the publication of this correction notice. Because this was made after submission to PubMed, PubMed Central, and other full-text repositories, the corrected article has also been resubmitted to those repositories.

## Appendix

Multimedia Appendix 1 Plain language summary.

