# Supplementary material for: The Patient Experience of Acute Lymphoblastic Leukemia and Its Treatment: Social Media Review
Source: JMIR Cancer. 2023 May 1;9:e39852. doi: 10.2196/39852 (PMC10186186; doi:10.2196/39852)
Supplement: Multimedia Appendix 1 [file cancer_v9i1e39852_app1.docx]

Appendix 1: Social Media Data Sources

| SM Data Source ID | SM Data Source Type | SM Data Source | SM Data Source Title | SM Data Source Website Link |
| --- | --- | --- | --- | --- |
| V_01 | Video | Patient Power | I Survived Acute Lymphoblastic Leukemia: Now What? | https://patientpower.info/acute-lymphoblastic-leukemia/living-with-acute-lymphoblastic-leukemia/i-survived-acute-lymphoblastic-leukemia-now-what |
| V_02 | Video | YouTube | Cancer Stories - André | https://www.youtube.com/watch?v=NlIQK-HvGD0 |
| V_03 | Video | YouTube | CAR-T therapy gives patient options after cancer returns | https://www.youtube.com/watch?v=9a08GOUvQX4 |
| V_04 | Video | YouTube | Matt May's Survivor Story | https://www.youtube.com/watch?v=g7vd01fnJ64 |
| V_05 | Video | YouTube | Vaughn Scott \| Acute Lymphoblastic Leukaemia (ALL) \| Spot Leukaemia | https://www.youtube.com/watch?v=rrkwMr5cz2c |
| V_06 | Video | YouTube | Late 20s Cancer Patient Doesn't Let Cancer Stop Him | https://www.youtube.com/watch?v=t_EmKTRT4FQ |
| V_07 | Video | YouTube | Alexandra Simpson \| Acute Lymphoblastic Leukaemia (ALL) \| Spot Leukaemia | https://www.youtube.com/watch?v=7u84HFUN3lc |
| V_08 | Video | YouTube | Sophie Sutton \| T-cell Acute Lymphoblastic Leukaemia (T-ALL) \| Spot Leukaemia | https://www.youtube.com/watch?v=wlE4g8V3X9U |
| V_09 | Video | YouTube | The Allana Guidry Story: A Mother's Bond | https://www.youtube.com/watch?v=w0zoOecQXY0 |
| V_10 | Video | YouTube | Tom Hunt - Acute Lymphoblastic Leukaemia (ALL) - Spot Leukaemia | https://www.youtube.com/watch?v=yA-PXpokD9E |
| V_11 | Video | YouTube | Sophie Wheldon \| B-cell acute lymphoblastic leukaemia (B-cell ALL) \| Spot Leukaemia | https://www.youtube.com/watch?v=hETia2HCGv8 |
| V_12 | Video | YouTube | Acute Lymphoblastic Leukaemia (ALL) stories: Brendan (Part 1) | https://www.youtube.com/watch?v=5PBqgHy99wc |
| V_13 | Video | YouTube | Milton Wright's Cancer Immunotherapy Story | https://www.youtube.com/watch?v=LjkofAY9bxI |
| V_14 | Video | YouTube | Acute lymphoblastic leukaemia stories: Brendan Wessely (Part 2 of 2) | https://www.youtube.com/watch?v=pNXsDY9TKXU |
| V_15 | Video | YouTube | My Life After Leukaemia \| Stand Up To Cancer | https://www.youtube.com/watch?v=dnJvkex0CNA |
| V_15_C_1 | Video Comment | YouTube | My Life After Leukaemia \| Stand Up To Cancer | https://www.youtube.com/watch?v=dnJvkex0CNA |
| V_15_C_2 | Video Comment | YouTube | My Life After Leukaemia \| Stand Up To Cancer | https://www.youtube.com/watch?v=dnJvkex0CNA |
| V_16 | Video | YouTube | Acute Lymphoblastic Leukemia Survivor: Lauren Johnson Shares Entire Treatment Experience | https://www.youtube.com/watch?v=Zct-6SlysX8 |
| V_16_C | Video Comment | YouTube | Acute Lymphoblastic Leukemia Survivor: Lauren Johnson Shares Entire Treatment Experience | https://www.youtube.com/watch?v=Zct-6SlysX8 |
| V_17 | Video | YouTube | Acute Lymphoblastic Leukemia (ALL): Michael’s Experience | https://www.youtube.com/watch?v=9xxZQm8U6Jk |
| V_18 | Video | YouTube | Jonjo's story: acute lymphoblastic leukaemia | https://www.youtube.com/watch?v=3hvn-_eV4Ww |
| V_18_C_1 | Video | YouTube | Jonjo's story: acute lymphoblastic leukaemia | https://www.youtube.com/watch?v=3hvn-_eV4Ww |
| V_18_C_2 | Video | YouTube | Jonjo's story: acute lymphoblastic leukaemia | [https://www.youtube.com/watch?v=3hvn-_eV4Ww](https://www.youtube.com/watch?v=3hvn-_eV4Ww&t=8s) |
| V_19 | Video | YouTube | Chloe Pinder - Acute Lymphoblastic Leukaemia patient story | https://www.youtube.com/watch?v=C9vAMJyCLps |
| V_20 | Video | YouTube | 60k for 60k - A Superhero Story | https://www.youtube.com/watch?v=Is1NH3Xr670 |
| V_21 | Video | YouTube | My Cancer Story. Leukemia. Cancer Vlog 1/X. | https://www.youtube.com/watch?v=WbIwpLig2A0 |
| V_22 | Video | YouTube | Acute Lymphoblastic Leukemia at 20 Years Old / Symptoms Leading To My Diagnosis | https://www.youtube.com/watch?v=wy0vxbR2sF0 |
| V_23 | Video | YouTube | Challenging the Odds of Recurrent Leukemia | https://www.youtube.com/watch?v=TkRguet-H7s |
| V_24 | Video | YouTube | Jeff Musser's Story | https://www.youtube.com/watch?v=P6PR9n3s3r4 |
| V_25 | Video | YouTube | Acute Lymphoblastic Leukemia Day 2 | https://www.youtube.com/watch?v=u7tr-oWkKss |
| V_26 | Video | YouTube | Day 2: No more kissing!!! | https://www.youtube.com/watch?v=ERuGlNJAmoU |
| V_27 | Video | YouTube | Day 4: I'm home for 2 weeks! | https://www.youtube.com/watch?v=zbpQstFZ_ec |
| V_28 | Video | YouTube | Day 3: Bone marrow biopsy | https://www.youtube.com/watch?v=dnGc8nCeD0w |
| V_29 | Video | YouTube | Day 6: THANK YOU TO EVERYONE!!!! | https://www.youtube.com/watch?v=dOkm5Bk3CbA |
| V_30 | Video | YouTube | Day 6: Fertility and Blood Levels Update | https://www.youtube.com/watch?v=ftVHxLJCw7w |
| V_31 | Video | YouTube | Day 17 - Pre Egg Retrieval and Start of Chemo Update | https://www.youtube.com/watch?v=VT-xP-Yh5mo |
| V_32 | Video | YouTube | Day 29 Round 2 of Chemo Done 👏🏻 | https://www.youtube.com/watch?v=vvhTSSEnGcY |
| V_33 | Video | YouTube | Day 37 ✅ Third Round of Chemo ✅ | https://www.youtube.com/watch?v=H1MSvHrfA7I |
| V_34 | Video | YouTube | Day 53 - Bone Marrow Biopsy Results/Plan Moving Forward | https://www.youtube.com/watch?v=JIJIoH-OM_Q |
| V_35 | Video | YouTube | Day 75 - Preparation for Hospital Stay #2 (Stem Cell Transplant) | https://www.youtube.com/watch?v=TLAlz5eQcMs |
| V_36 | Video | YouTube | Summer Smith \| Acute Lymphoblastic Leukaemia (ALL) \| Spot Leukaemia | https://www.youtube.com/watch?v=nl4xwjPYjrc |
| V_37 | Video | YouTube | Venessa Taylor - Acute Lymphoblastic Leukaemia - Spot Leukaemia | https://www.youtube.com/watch?v=erRL3KLA1QQ |
| V_38 | Video | Patient Power | Young Adult With ALL: Why I Chose IV Therapy | <https://patientpower.info/acute-lymphoblastic-leukemia/patient-stories/young-adult-with-all-why-i-chose-iv-therapy#read-transcript-anchor> |
| V_39 | Video | Patient Power | Confidence in Your Care: A Team Approach to Treating ALL | <https://patientpower.info/acute-lymphoblastic-leukemia/treatments/confidence-in-your-care-a-team-approach-to-treating-all> |
| V_40 | Video | Patient Power | An Olympic Athlete’s Take on His Acute Lymphoblastic Leukemia Treatment | <https://patientpower.info/acute-lymphoblastic-leukemia/patient-stories/an-olympic-athletes-take-on-his-acute-lymphoblastic-leukemia-treatment> |
| B_01 | Blog | The Patient Story | William’s B-cell Acute Lymphoblastic Leukemia Story | https://www.thepatientstory.com/cancers/leukemia/acute-lymphoblastic-leukemia-all/william-yank/ |
| B_02 | Blog | The Patient Story | Ciara’s Acute Lymphoblastic Leukemia Story | https://www.thepatientstory.com/cancers/leukemia/acute-lymphoblastic-leukemia-all/ciara-toth/ |
| B_03 | Blog | The Patient Story | Christine’s Relapsed T-Cell Acute Lymphoblastic Leukemia Story | https://www.thepatientstory.com/cancers/leukemia/acute-lymphoblastic-leukemia-all/christine-mills/ |
| B_04 | Blog | The Patient Story | Veronica’s Refractory Ph- B-Cell Acute Lymphoblastic Leukemia Story | https://www.thepatientstory.com/cancers/leukemia/acute-lymphoblastic-leukemia-all/veronica-balanza/ |
| B_05 | Blog | The Patient Story | Evan’s Acute Lymphoblastic Leukemia Story | https://www.thepatientstory.com/cancers/leukemia/acute-lymphoblastic-leukemia-all/evan-lessler/ |
| B_06 | Blog | The Patient Story | Lauren’s Acute Lymphoblastic Leukemia Story | https://www.thepatientstory.com/cancers/leukemia/acute-lymphoblastic-leukemia-all/lauren-johnson/ |
| B_07 | Blog | Leukaemia Care | Maria Pugh | https://www.leukaemiacare.org.uk/support-and-information/latest-from-leukaemia-care/inspirational-stories/maria-pugh/ |
| B_08 | Blog | Leukaemia Care | Sophie Wheldon | https://www.leukaemiacare.org.uk/support-and-information/latest-from-leukaemia-care/inspirational-stories/sophie-wheldon/ |
| B_09 | Blog | Leukaemia Care | Vaughn Scott | https://www.leukaemiacare.org.uk/support-and-information/latest-from-leukaemia-care/inspirational-stories/vaughn-scott/ |
| B_10 | Blog | Leukaemia Care | Sophie Sutton | https://www.leukaemiacare.org.uk/support-and-information/latest-from-leukaemia-care/inspirational-stories/sophie-sutton/ |
| B_11 | Blog | Leukaemia Care | Summer Smith | https://www.leukaemiacare.org.uk/support-and-information/latest-from-leukaemia-care/inspirational-stories/summer-smith/ |
| B_12 | Blog | Leukaemia Care | Alexandra Simpson | https://www.leukaemiacare.org.uk/support-and-information/latest-from-leukaemia-care/inspirational-stories/alexandra-simpson/ |
| B_13 | Blog | Leukaemia Care | Venessa Taylor | https://www.leukaemiacare.org.uk/support-and-information/latest-from-leukaemia-care/inspirational-stories/venessa-taylor/ |
| B_14 | Blog | Leukaemia Care | Tom Hunt | https://www.leukaemiacare.org.uk/support-and-information/latest-from-leukaemia-care/inspirational-stories/tom-hunt/ |
| B_15 | Blog | Leukaemia Care | Gary Bowman | https://www.leukaemiacare.org.uk/support-and-information/latest-from-leukaemia-care/inspirational-stories/gary-bowman/ |
| B_16 | Blog | Leukaemia Care | Kelly Chambers | https://www.leukaemiacare.org.uk/support-and-information/latest-from-leukaemia-care/inspirational-stories/kelly-chambers/ |
| B_17 | Blog | Leukaemia Care | Chloe Pinder | https://www.leukaemiacare.org.uk/support-and-information/latest-from-leukaemia-care/inspirational-stories/chloe-pinder/ |
| B_18 | Blog | Leukaemia Care | Ryan Jay | https://www.leukaemiacare.org.uk/support-and-information/latest-from-leukaemia-care/inspirational-stories/ryan-jay/ |
