# Supplementary material for: The Patient Experience of Acute Lymphoblastic Leukemia and Its Treatment: Social Media Review
Source: JMIR Cancer. 2023 May 1;9:e39852. doi: 10.2196/39852 (PMC10186186; doi:10.2196/39852)
Supplement: Multimedia Appendix 2 [file cancer_v9i1e39852_app2.pdf]

Plain Language Summary

Please note that this summary only contains information from the full scientific article:

View Scientific Article

Understanding the experiences of people with acute lymphoblastic leukemia using social media

Acute lymphoblastic leukemia
<uh-KYOOT LIM-foh-BLAS-tik loo-KEE-mee-uh>

ALL
<A-ell-ell>

Chemotherapy
<KEE-moh-THAYR-uh-pee>

Lymphoblast
<LIM-foh-BLAST>

Neutrophil
<NOO-troh-fil>

Date of summary: May 2023

The full title of this article: The patient experience of acute lymphoblastic leukemia and its treatment: social media review

Key takeaways

- Social media can be used for understanding the experiences of people living with acute lymphoblastic leukemia (ALL for short) who choose to share their thoughts online.
- Symptoms of ALL and side effects of treatment appear to affect different aspects of people's day-to-day lives.
  - A side effect is something (expected or unexpected) that you feel was caused by a medicine or treatment you take.
  - Symptoms of ALL appear to affect people's physical functioning the most, including activities of daily living and their ability to work.
  - Side effects of treatment appear to affect people's mental health the most.

The purpose of this plain language summary is to help you understand the findings from a recent study, which involved a review of social media.

- This summary reports the results of a single study. The results of this study may differ from those of other studies. Health professionals should make treatment decisions based on all available evidence, not on the results of a single study.
- The results of this study are based on information shared on social media by people living with ALL. These results may not fully represent the views of all people living with ALL.

More information can be found in the scientific article of this study, which you can access here: View Scientific Article

Additional information

More information can be found in the scientific article of this study, which you can access here:

View Scientific Article

Acute lymphoblastic leukemia
<uh-KYOOT LIM-foh-BLAS-tik loo-KEE-mee-uh>

ALL
<A-ell-ell>

Chemotherapy
<KEE-moh-THAYR-uh-pee>

Lymphoblast
<LIM-foh-BLAST>

Neutrophil
<NOO-troh-fil>

What did this study look at?

- ALL is a type of blood cancer.
  - For people with ALL, the body makes too many lymphoblasts. These are a type of white blood cell, released from bone marrow before they have fully developed.
    - All the blood cells in the body develop from stem cells produced by bone marrow. This is a spongy material found inside bones.
  - As the number of lymphoblasts increases, the number of red blood cells (which carry oxygen around the body) and platelets (which help stop bleeding) decreases.
    - This can cause tiredness, shortness of breath, and an increased chance of bleeding.
  - Lymphoblasts are also less effective than fully developed white blood cells at fighting bacteria and viruses, which makes people more vulnerable to infection.
- ALL and its treatment continue to negatively affect people's day-to-day lives.
- Understanding the impact of ALL and its treatment on people's lives will help researchers to develop treatments that meet people's needs.
- In this study, researchers looked at social media posts in English on publicly available websites. This is known as patient-reported information. Researchers wanted to understand what people were saying online about:
  - Their experiences of living with ALL.
  - Their experiences of treatment.
  - How ALL and its treatment affected their day-to-day lives.

How was this study done?

In October 2020, researchers searched

6 websites

including 5 patient support websites and YouTube, for social media posts from people living with ALL

CURE®
Leukaemia Care
Patient Power
Patients Rising
The Patient Story
YouTube

Researchers found

935 original social media posts

containing information from people living with ALL from 3 patient support websites and YouTube

Leukaemia Care
Patient Power
The Patient Story
YouTube

63 posts

most of which were uploaded in 2018 or later, mentioned ALL and its treatment

- 40 videos
- 5 video comments
- 18 blog posts

Additional information

More information can be found in the scientific article of this study, which you can access here:

View Scientific Article

Acute lymphoblastic leukemia
<uh-KYOOT LIM-foh-BLAS-tik loo-KEE-mee-uh>

ALL
<A-ell-ell>

Chemotherapy
<KEE-moh-THAYR-uh-pee>

Lymphoblast
<LIM-foh-BLAST>

Neutrophil
<NOO-troh-fil>

- The 63 posts were from 41 people with a self-reported diagnosis of ALL.
  – They were age 18 years or older.
  – Around half (21 people) were male.
- Based on available information in their profiles:
  – 19 people were from the United States.
  – 15 people were from the United Kingdom.
  – 1 person was from Australia.
  – 1 person was from Canada.
  – 1 person was from South Africa.
  – 4 people's location could not be identified.

What were the results of this study?

Experience of ALL symptoms

- People described the most common symptoms before treatment.

Out of 41 people:

20 people reported feeling tired and lacking energy — 49%

13 people reported shortness of breath — 32%

12 people reported bruising — 29%

Symptoms had an impact on people's physical functioning:

- 5 people said they had trouble climbing stairs or walking uphill — 12%
- 4 people said they had trouble getting out of bed — 10%
- 3 people said they had trouble walking short distances — 7%

Additional information

More information can be found in the scientific article of this study, which you can access here:

View Scientific Article

Acute lymphoblastic leukemia
<uh-KYOOT LIM-foh-BLAS-tik loo-KEE-mee-uh>

ALL
<A-ell-ell>

Chemotherapy
<KEE-moh-THAYR-uh-pee>

Lymphoblast
<LIM-foh-BLAST>

Neutrophil
<NOO-troh-fil>

- People described the impact of ALL symptoms on day-to-day lives.

Out of 41 people:

26 people said that ALL symptoms affected their personal relationships — 63%

- 5 people said that relationships were strengthened — 12%
- 2 people said that they lost touch with friends and their relationships with family members changed — 5%

25 people said that ALL symptoms affected their mental health — 61%

- 4 people said they were anxious over ALL returning — 10%
- 4 people said they were fearful of the future, including dying — 10%
- 3 people said they experienced low mood — 7%
- 2 people said they were lonely — 5%
- 2 people said that life was uncertain with ALL — 5%

16 people said that ALL symptoms affected their ability to work — 39%

11 people said that ALL symptoms affected their ability to do daily tasks, such as self-care, shopping, and leisure activities — 27%

2 people also said that symptoms affected their social lives — 5%

Additional information

More information can be found in the scientific article of this study, which you can access here:

View Scientific Article

Acute lymphoblastic leukemia
<uh-KYOOT LIM-foh-BLAS-tik loo-KEE-mee-uh>

ALL
<A-ell-ell>

Chemotherapy
<KEE-moh-THAYR-uh-pee>

Lymphoblast
<LIM-foh-BLAST>

Neutrophil
<NOO-troh-fil>

Experience of treatment

- Nearly half of the people (20 out of 41; 49%) had received 2 or more types of treatment.
  - 31 people (76%) had received chemotherapy.
  - 11 people (27%) had received a bone marrow transplant.
    - Bone marrow transplant is a procedure where people receive healthy stem cells to replace their own stem cells destroyed by high doses of chemotherapy or radiation.
- People described the most common treatment side effects.

Out of 41 people:

11 people reported feeling tired and lacking energy — 27%

11 people reported hair loss — 27%

9 people reported feeling sick — 22%

- People said that these side effects often lasted for a long time.
- Treatment side effects affected people's day-to-day activities, such as using their hands, showering, and eating.
  - One person was concerned that treatment side effects would affect their ability to have children in the future.
- Treatment side effects negatively affected people's mental health.
  - One person felt that treatment for ALL was worse than the condition itself.

- **3 key themes emerged from the analysis of social media posts related to the experience of treatment:**
  - Treatment in hospital.
  - Treatment expectations and wishes.
  - Making treatment decisions.

Additional information

More information can be found in the scientific article of this study, which you can access here:

View Scientific Article

Acute lymphoblastic leukemia
<uh-KYOOT LIM-foh-BLAS-tik loo-KEE-mee-uh>

ALL
<A-ell-ell>

Chemotherapy
<KEE-moh-THAYR-uh-pee>

Lymphoblast
<LIM-foh-BLAST>

Neutrophil
<NOO-troh-fil>

Additional information

More information can be found in the scientific article of this study, which you can access here:

View Scientific Article

Treatment in hospital

Out of 41 people:

11 people discussed their experience of receiving treatment in hospital — 27%

- 4 people felt that it reduced their independence — 10%
- 4 people felt that it reduced their social life — 10%
- 1 person felt it was a source of anxiety, because they were worried about looking after their children while in hospital
- 4 people felt it gave them a sense of safety — 10%
  - These people appreciated access to regular care and having their treatment managed by nurses

Treatment expectations and wishes

Out of 41 people:

15 people discussed their treatment expectations and wishes — 37%

- 4 people said that they expected treatment side effects — 10%
- 3 people said that side effects were not always as bad as they had imagined — 7%

- People living with ALL preferred treatment that had the least possible impact on their day-to-day lives.
  - They wanted to maintain an independent lifestyle with as few side effects of treatment as possible.

Acute lymphoblastic leukemia
<uh-KYOOT LIM-foh-BLAS-tik loo-KEE-mee-uh>

ALL
<A-ell-ell>

Chemotherapy
<KEE-moh-THAYR-uh-pee>

Lymphoblast
<LIM-foh-BLAST>

Neutrophil
<NOO-troh-fil>

Making treatment decisions

Out of 41 people:

9 people described how they made treatment decisions — 22%

7 people said they relied on their doctors to help them make treatment decisions — 17%

Some people said that the shock of their diagnosis meant they were unaware of treatment options, and they did not necessarily take everything in

3 people said that their treatment decisions were influenced by parents — 7%

Links between symptoms and side effects on people's day-to-day lives

- Researchers looked at possible links between symptoms and side effects of treatment on people's day-to-day lives.
- The symptoms of ALL appeared to have the most impact on people's physical functioning, daily activities, and ability to work.
- The side effects of treatment appeared to have more of an impact on people's mental health than their physical functioning.
- Some side effects of treatment were associated with changes in eating habits linked to weight loss. These included:
  - Low levels of neutrophils, which are a type of white blood cell.
  - Changes in taste.
  - Feeling sick.
  - Mouth sores.
- 3 people thought these side effects were due to chemotherapy.
- 1 person thought these side effects were linked to a stem cell transplant.
- Physical limitations appeared to have a negative influence on people's day-to-day lives, impacting their daily activities, work, travel, mental health, and relationships.

Additional information

More information can be found in the scientific article of this study, which you can access here:

View Scientific Article

Acute lymphoblastic leukemia
<uh-KYOOT LIM-foh-BLAS-tik loo-KEE-mee-uh>

ALL
<A-ell-ell>

Chemotherapy
<KEE-moh-THAYR-uh-pee>

Lymphoblast
<LIM-foh-BLAST>

Neutrophil
<NOO-troh-fil>

What were the main conclusions reported by the researchers?

- This is considered to be the first piece of research using social media to understand people's experiences of ALL and its treatment.
- Symptoms of ALL and side effects of treatment appeared to affect different aspects of people's day-to-day lives.
  - ALL symptoms mostly affected people's physical functioning, activities of daily living, and ability to work.
  - Side effects of treatment appeared to affect people's mental health the most.
- Receiving treatment in the hospital was associated with reduced independence and socializing. However, some people appreciated the sense of security provided when receiving treatment in the hospital.
- Overall, people living with ALL wanted treatments that would have the least possible impact on their day-to-day lives.

Who sponsored this study?

Pfizer Inc.

235 East 42nd Street, NY, NY 10017

Phone (United States): +1 212-733-2323

Pfizer thanks everyone who contributed to this research.

Scientific Article

Crawford R, Sikirica S, Morrison R, et al. The patient experience of acute lymphoblastic leukemia and its treatment: social media review. *JMIR Cancer* 2023;9:e39852. doi: 10.2196/39852

Acknowledgements

Writing support for this summary was provided by Simon R. Stones, PhD, ISMPP CMPP™, of Engage Scientific Solutions, and was funded by Pfizer. The original authors of the full article were involved in preparing this summary.

Additional information

More information can be found in the scientific article of this study, which you can access here:

View Scientific Article
